# Investigating alveolar macrophages in an human ex vivo precision‐cut lung slice model of SARS‐CoV‐2 infection using Raman spectroscopy—A case study

**DOI:** 10.1002/ctm2.70453

**Published:** 2025-08-31

**Authors:** Max Naumann, Franziska Hornung, Simone Eiserloh, Astrid Tannert, Antje Häder, Rustam R. Guliev, Tim Sandhaus, Stefanie Deinhardt‐Emmer, Ute Neugebauer

**Affiliations:** ^1^ Leibniz Institute of Photonic Technology, Member of Leibniz Health Technologies Member of the Leibniz Centre for Photonics in Infection Research (LPI) Jena Germany; ^2^ Center for Sepsis Control and Care (CSCC) Jena University Hospital Jena Germany; ^3^ Institute of Medical Microbiology Jena University Hospital Jena Germany; ^4^ Clinic for Heart and Thoracic Surgery Jena University Hospital Jena Germany; ^5^ Department of Anesthesiology and Intensive Care Medicine Jena University Hospital Jena Germany; ^6^ Institute of Physical Chemistry and Abbe Center of Photonics Friedrich Schiller University Jena Germany

**Keywords:** alveolar macrophages, delta, human precision‐cut lung slice model, label‐free phenotyping, omicron, phenotype plasticity, principal component analysis, Raman spectroscopy, SARS‐CoV‐2, single‐cell analysis

## Abstract

**Background:**

Alveolar macrophages (AMs) are crucial innate immune cells that play important roles during infection with severe acute respiratory syndrome coronavirus type 2 (SARS‐CoV‐2). Ex vivo human precision‐cut lung slices (PCLSs) are well‐suited models to study immune reactions and biochemical changes within host cells as well as to follow functional macrophage phenotype plasticity within complex tissue environment. Raman spectroscopy emerged in recent years as a powerful method for label‐free cell characterization.

**Methods:**

Human PCLSs from one donor were infected with either the SARS‐CoV‐2 delta or omicron variant. Immunofluorescence microscopy localized AMs and virus particles. Cytokine levels of interferon‐gamma (IFN‐γ) and interleukin 18 (IL‐18) were quantified. The lung slice model was optimized for label‐free Raman spectroscopic imaging and for the characterization of single AMs within the three‐dimensional structure of the PCLS model.

**Results:**

Fluorescence microscopy confirmed the location of AMs and virus particles within the PCLS model. Raman spectroscopic imaging generated false‐colour images, revealing distinct spectroscopic differences between AMs in the uninfected control PCLS model and those in PCLS models infected with SARS‐CoV‐2. These differences included variations in intracellular RNA, carotenoid, triacyl glyceride, and glucose levels, consistent in interpretation with cytokine quantification data. A linear discriminant analysis (LDA) classification model achieved an 83% accuracy in distinguishing cells from infected lung slices from those of the uninfected controls. The LDA loadings pointed to spectral bands that had been previously identified in an in vitro stimulation study of macrophages.

**Conclusions:**

Raman spectroscopy can characterize the cellular immune response and phenotype plasticity of AMs to infection with SARS‐CoV‐2 within a PCLS model in a label‐free and non‐invasive manner. The ability to distinguish cells from infected PCLSs from cells of the uninfected control PCLS based on intracellular biochemical changes highlights the potential of Raman spectroscopy as a powerful diagnostic tool in immunology and clinical diagnostics.

## Graphical abstract

Label‐free Raman spectroscopy is utilized for an in‐depth characterization of alveolar macrophages directly within intact precision‐cut human lung slices. Characteristic biochemical changes in individual alveolar macrophages upon ex vivo infection with SARS‐CoV‐2 virus are revealed and enable a reliable statistical differentiation of single macrophages from SARS‐CoV2 infected tissue slices from those of uninfected controls.

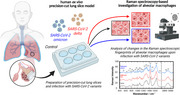

## 1 BACKGROUND

Alveolar macrophages (AMs) are crucial innate immune cells in the lung, maintaining homeostasis and orchestrating inflammatory responses.^1^, ^2^ Recent advancements in single cell sequencing and imaging techniques have revealed the cellular and functional diversity of AMs, highlighting their adaptability in health and disease.^3^, ^4^ During acute lung injury, AMs play a central role in initiating and resolving inflammation, making them attractive therapeutic targets.^2^ However, modulating AM function presents challenges, as anti‐inflammatory phenotypes may conflict with pro‐inflammatory and antimicrobial responses. Hence, the identification of cellular key indicators that determine AM fate decisions is crucial for harnessing their therapeutic potential while maintaining essential host defence functions.^2^, ^3^

Recent research also highlights this crucial role of AMs during severe acute respiratory syndrome coronavirus type 2 (SARS‐CoV‐2) infection and in lung pathology. In general, AMs are responsible for recognition of invading pathogens and damaged or dead cells, their clearance via phagocytosis, but also for cytokine production. Although the polarization concept of macrophages itself is still subject of ongoing research,^5^ it was shown that the polarization state of AMs significantly influences viral spread, with M1‐like AMs facilitating and M2‐like AMs limiting viral propagation.^6^ While AMs efficiently phagocytose SARS‐CoV‐2, the acidic endosomal pH (especially in M1‐type macrophages) enables SARS‐CoV‐2 to leave endosomes and initiate virus replication in the cytosol.^7^ The omicron variant harbours many basic amino acid mutations in the spike protein, which facilitate endosomal escape also at less acidic pH and contribute to the ultrafast spread of the omicron variant.^7^ However, the understanding of AM responses to SARS‐CoV‐2 variants at the phenotypic level is still limited, and there is a need for new and advanced analytical techniques for such specific investigation, especially also to enable the identification of new therapeutic targets for SARS‐CoV‐2 treatment strategies.^7^, ^8^

Raman spectroscopy is emerging as a powerful diagnostic tool in biology and medicine, offering rapid, non‐destructive, and label‐free analysis of biological samples.^9^, ^10^, ^11^, ^12^ Thereby, monochromatic light is inelastically scattered at molecules within the focal volume, eventually resulting in unique spectra after the backscattered photons have been analysed for their wavenumber shift. The spectra summarize the information about the chemical composition and can be interpreted as spectroscopic fingerprint of a sample under specified conditions. This technique has shown promise in characterizing macrophage polarization, distinguishing between pro‐inflammatory M1 and anti‐inflammatory M2 phenotypes based on their biochemical profiles.^13^, ^14^, ^15^

The aim of this study is to explore the AM phenotype upon infection with SARS‐CoV‐2 variants using a human ex vivo precision‐cut lung slice (PCLS) models. Therefore, non‐invasive Raman spectroscopic single‐cell imaging followed by spectroscopic characterization of AMs was performed within intact tissue slices.

## 2 METHODS

### 2.1 Human ex vivo PCLS model

PCLSs were generated from human lung tissue following a previously established protocol.^16^ In summary, lung tissue was infused with a mixture of medium and agarose. Well‐perfused regions were selected, embedded in agarose and sectioned into slices of 300 µm thickness. These slices were then cultured in well plates with DMEM/F12 (Gibco, Thermo Fisher Scientific) under conditions of 37°C and 5% CO_2_ for further experimentation. Several slices were used as technical replicates from the same donor. Experiments utilized lung tissue from one healthy male donor aged 35 with no known underlying diseases, no preconditions and explicitly no antibiotic pre‐treatment. This research was approved by the local ethics board (approval numbers: 2018‐1263, 2020‐1894 and 2020‐1773).

### 2.2 SARS‐CoV‐2 propagation and infection

For all experiments, SARS‐CoV‐2 isolates obtained from respiratory specimens of different patients were utilized (ethics approval from Jena University Hospital, no.: 2018‐1263) as published previously.^16^ To propagate the isolates, Vero‐76 cells were seeded in T25 cell culture flasks, washed 12 h later, and infected with 200 µL of filtered viral isolate obtained from patient samples (sterile syringe filter, 0.2 µm pore size) in the presence of Panserin 401 (PanBiotech). Cytopathic effects became visible after 5 days. The cell culture supernatants were harvested and centrifuged at 4°C with 5000 rpm for 10 min to obtain cell‐free virus solution that was stored at −40°C.

### 2.3 Infection of PCLSs

After 48 h of adjustment to conditions in the well plate, infection of the PCLS model was performed with SARS‐CoV‐2 delta and omicron variants that were propagated from patient samples. A final concentration of 5 × 10^5^ PFU/mL was adjusted with cultivation medium. PCLS were washed with phosphate‐buffered saline (PBS, Gibco, Thermo Fisher Scientific) and incubated with the virus for 2 h. Then, the supernatants were aspirated and replaced with DMEM/F12 (Gibco, Thermo Fisher Scientific). After 2 days of infection, PCLS samples were fixed for imaging with 4% paraformaldehyde (Roti Histofix, Carl Roth) over night, washed with 1× PBS and stored at 4°C until Raman spectroscopic measurements. For cytokine quantification, some PCLS samples were kept also until day 4. Uninfected PCL slices served as controls in the study. Selection of post‐infection time points was based on our previous study.^16^ Around day 2 post‐infection, typically a peak of viral load is observed, corresponding to the early infection phase,^17^, ^18^ which was in the focus of the study. For cytokine quantification, additional day 4 was included to also capture the main phase of infection.^17^

### 2.4 Cytokine quantification

Cytokine levels (interferon gamma [IFN‐γ] and interleukin 18 [IL‐18]) in the supernatants of PCLS were measured using the LEGENDplex Human Inflammation Panel 1 Kit (BioLegend) following the manufacturer's instructions. Samples were measured with a FACS Symphony A1 flow cytometer (BD Biosciences) and analysed with the LEGENDplex Data Analysis Software (v2024‐06‐15). Data were collected from three independent PCLS per condition.

### 2.5 Immunofluorescence staining and imaging

**FIGURE 1:**
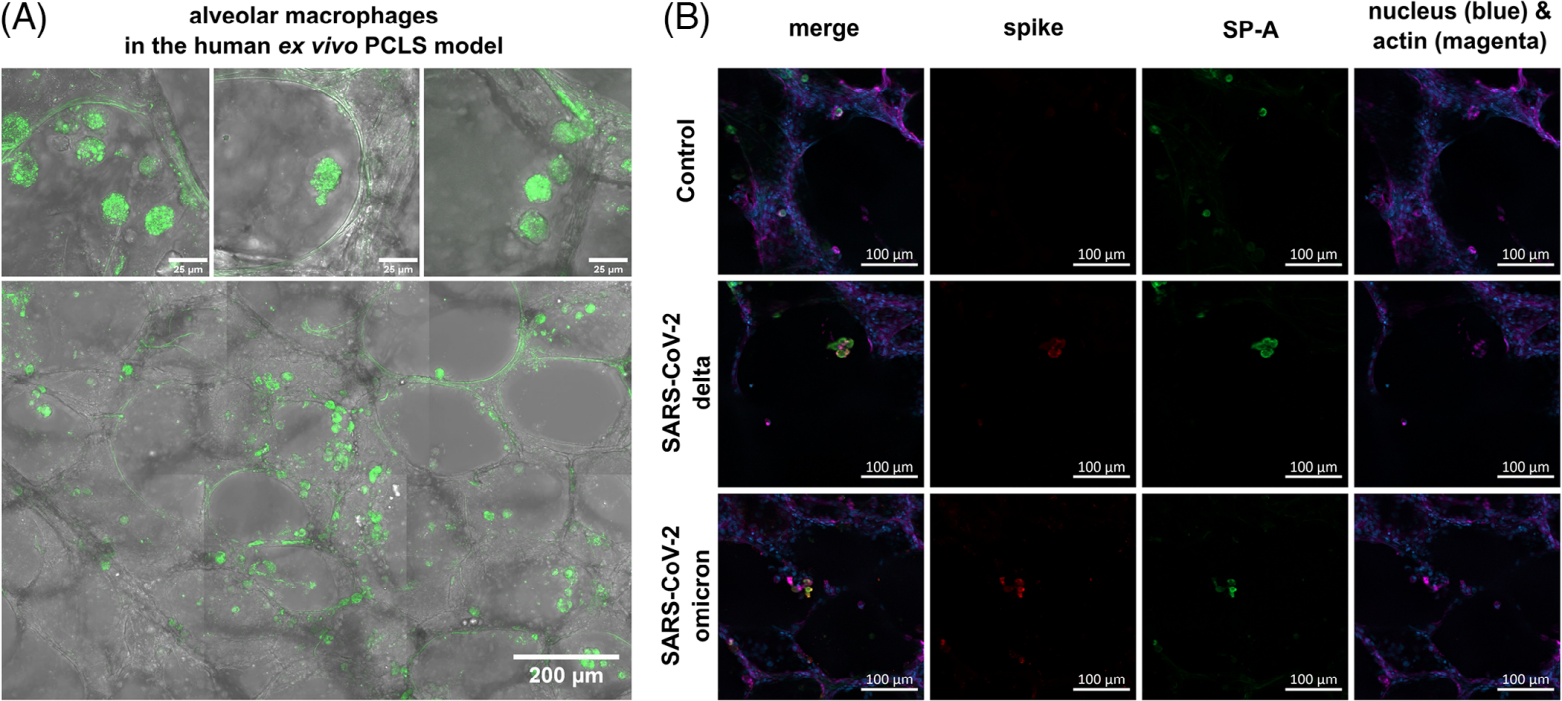
Localization of alveolar macrophages and SARS‐CoV‐2 virus within the alveoli of human ex vivo precision‐cut lung slice (PCLS) models. (A) Fluorescence images of cells labelled with anti‐CD11b PE‐conjugated antibodies (green) at 63× (top row) and 20× (bottom) magnification. Alveolar macrophage can be recognized based on fluorescence staining, their spherical shape and size (diameter of 20–25 µm) as well as their location within the alveoli. (B) Fluorescence‐labelled spike proteins (red) indicates successful infection of the lung tissue. Surfactant protein A (SP‐A, green) visualizes alveolar type II epithelial cells. Cell nuclei (blue) and actin (magenta) are stained to orient within the tissue slice. Co‐localization of spike protein within the round alveolar macrophage (AM) within the alveolar space indicates the presence of viral material also in alveolar macrophages.

For localization of AMs (Figure 1A), PCLSs were placed on coverslips, fixed with 4% paraformaldehyde (PFA, Sigma Aldrich) for 15 min at 37°C, and stored in DPBS (Thermo Fisher Scientific) at 4°C until staining. Permeabilization was performed using 1% Triton‐X 100 (Carl Roth) for 15 min at room temperature (RT), followed by blocking with 3% bovine serum albumin (BSA; Carl Roth) in DPBS for 30 min at RT. The PE‐conjugated IgG2bκ antibody REAfinity anti‐human CD11b (Miltenyi Biotec, Cat #130‐113‐797) diluted 1:50 was used for staining. Imaging was conducted using a confocal laser scanning microscope (LSM 780 META, Carl Zeiss) with a Plan‐Apochromat 20×/0.8 NA objective (Carl Zeiss) or a 63×/1.4 NA oil immersion objective (Carl Zeiss). PE was excited using a 561 nm laser line and emission was detected in the range of 571–695 nm.

For immunostaining of SARS‐CoV‐2 virus (Figure 1B), PCLSs were fixed, permeabilized and blocked as described above. Antibodies against SARS‐CoV‐2 spike antibody (GeneTex, Cat #GTX635807) and Surfactant Protein A (SP‐A) (Novus Biologicals, Cat # NBP212928) were used. Detailed information about primary and secondary antibodies are listed in Table 1.

**TABLE 1:** Antibodies used for immunofluorescence staining of PCLS to visualize CD11b‐positive immune cell populations and successful infection.

Target protein	Primary antibody	Dilution of primary antibody	Secondary antibody	Dilution of secondary antibody	Purpose
CD11b	PE‐conjugated IgG2bκ antibody REAfinity anti‐human CD11b (Miltenyi Biotec, Cat #130‐113‐797)	1:50	–	–	Visualize CD11b‐positive cells (esp. macrophages)
SARS‐CoV‐2 spike	SARS‐CoV‐2 spike antibody (GeneTex, Cat #GTX635807)	1:200	Cy3‐conjugated AffiniPure Donkey Anti‐Rabbit IgG antibody (Jackson ImmunoResearch, Cat #711‐165‐152)	1:500	Visualize SARS‐CoV‐2 infection by staining spike protein
Surfactant Protein A	Surfactant Protein A Antibody (Novus Biologicals, Cat # NBP212928)	1:100	Alexa Fluor 488 AffiniPure Donkey Anti‐Goat IgG (Jackson Immuno Research, Cat #705‐545‐147)	1:500	Identify and delineate alveolar epithelial type II cells, which are a known target for SARS‐CoV‐2 infection in the lung

Abbreviation: PCLS, precision‐cut lung slice.

To visualize the cells in the PCLSs, actin was stained using Alexa Fluor Plus 647 Phalloidin staining solution (Invitrogen, Cat#A30107) diluted 1:400. The coverslips were incubated for 1 h at room temperature in blocking buffer and then mounted using DAPI Fluoromount‐G (Southern Biotech) to stain cell nuclei. Imaging was conducted using an AxioObserver Z.1 + Apotome 2 microscope (Carl Zeiss) with an EC Plan‐Neofluar 10×/.30 M27. The images were acquired using separate channels for DAPI (Ex 335–383 nm/Em 420–470 nm), Cy3 (Ex 538–562 nm/Em 570–640 nm), Cy5 (Ex 625–655 nm/Em 665–715 nm), and GFP (Ex 450–490 nm/Em 500–550 nm) detection.

### 2.6 Raman spectroscopic imaging

For Raman spectroscopic imaging an *α*300 Raman spectrometer (WITec) equipped with a 785 nm laser (200 mW in the object plane; Toptica GmbH) and a 60×/1.0 NIR Apo water immersion objective (Nikon GmbH) was used. An optical fibre (Ø 100 µm) was applied to guide the backscattered Raman light to a spectrometer. The signal was detected by a back‐illuminated deep depletion CCD camera (DV401A‐BV‐352 cooled to −60°C, Andor). Brightfield overview images and subsequent Raman scans were performed on the same device. Thus, a globally defined coordinate system was established for each PCLS sample within the Raman microscope. This coordinate system was used for both imaging modalities (brightfield and Raman) and did not change.

For imaging of AMs, brightfield overview images were recorded and AMs were manually selected within the coordinate system of the device. For selecting AMs, we were relying on both, characteristics in location as well as morphological characteristics (as previously also verified in CD11b‐stained fluorescence images). Those criteria were in particular:

1. Location criteria: we have chosen cells that are inside the airspaces (the alveoli) and only loosely attached to the alveolar epithelium (see Figure S2).
2. Morphological criteria: we have selected cells that have round to oval shape and are relatively large (around 15–20 µm in diameter). In addition, the selected cells showed a granular cytoplasm.

The optimal focal plane for each image scan within the tissue was selected by automated focusing based on the best signal‐to‐noise ratio within the (1450 ± 50) cm^−1^ Raman spectral region. Spectral imaging was done using a spatial step size of 0.5 µm in an area of 15–30 µm in both *x* and *y* direction and an integration time of 1 s per spectrum for most of the cells. In total, 15 cells (5 from each condition: uninfected control, infection with SARS‐CoV‐2 delta variant and infection with SARS‐CoV‐2 omicron variant) were measured yielding 10 000–18 000 spectra per condition (in total: 46 000 spectra, Table S1).

### 2.7 Statistical analysis of spectroscopic data

Pre‐processing and analysis of Raman spectra was done using R (version 4.1) and the following packages: ggplot2,^19^ gridExtra^20^ for visualization, MASS^21^ for LDA calculation, hyperSpec^22^ and dplyr^23^ for data import and manipulation, matrixStats^24^ for optimized matrix operations, and unmixR^25^ for N‐FINDR algorithm. Outlier spectra and background spectra were excluded using kmeans clustering. Cosmic ray noise was automatically corrected using the algorithm described in Ryabchykov et al.^26^ The spectral silent region between 1800 and 2700 cm^−1^ was removed and the baseline was corrected using SNIP algorithm^27^ with 50 iterations. Eventually, spectra were mean normalized. After spectral preprocessing, 22 194 Raman spectra from 15 cells were used in subsequent statistical analysis (Table S1).

False‐colour Raman images were generated using the N‐FINDR algorithm for endmember (i.e. pure component) extraction and NNLS (Non‐Negative Least Squares) for abundance calculation. Details on the algorithm can be found in the original work^27^ and the respective R package.^24^ To avoid the influence of remaining outlier and background spectra, 5–10 endmembers were chosen and manually assigned to cellular compartments and biomolecules such as nuclei with DNA, cell membrane with phospholipids, cytosol with proteins, lipids and carbohydrates for each cell investigated.

In order to build a discrimination model based on Raman spectroscopic data, a principal component analysis (PCA)^28^ followed by linear discriminant analysis (LDA)^29^, ^30^ was done. Average spectra per cell were chosen as input data. The model was cross‐validated, leaving out one cell of each group. Model accuracy metrics were calculated based on testing predictions.

For quantitative analysis of the pre‐processed Raman spectra with respect to specific biochemical compounds within each investigated AM, Raman intensities within critical spectral regions were summarized and visualized in box plots. The spectral regions as well as the Raman bands annotated in the spectra were selected based on previously published data^31^, ^32^, ^33^, ^34^, ^35^, ^36^ and are summarized in Table S2.

OriginPro 2025 (version 10.2.0.188) software was used to generate the final graphical representation of the data.

## 3 RESULTS

### 3.1 Fluorescence microscopy‐based characterization of the human ex vivo PCLS model

For successful Raman spectroscopic imaging of AMs in the ex vivo PCLS model, the position of the cells within the alveolar lung structures was validated using confocal laser scanning microscopy (cLSM, Figure 1A). AMs (as well as dendritic cells, monocytes and granulocytes) were labelled with anti‐CD11b PE‐conjugated antibodies. Among the labelled cells, AMs could be identified based on their location within the alveolar space. Many alveoli contained spherical AMs with granular cytoplasm. The granularity could originate from dust, invading organisms and particles or other ingested debris. The fixed cells were 20–25 µm in diameter and appeared to be considerably larger than other cells within the tissue model. Size and granularity are consistent with published data.^37^

Immunofluorescence labelling of viral spike proteins visualized the successful infection of the lung tissue. Surfactant protein A (SP‐A) was included to identify alveolar type II epithelial cells, which are established targets of SARS‐CoV‐2 infection. In addition, SP‐A expression allows to assess the structural integrity of the alveolar epithelium within infected tissue sections (Figure 1B). Co‐localization of spike protein within the round AM within the alveolar space indicates the presence of viral material also in AMs (Figure 1B).

Knowing the location and microscopic appearance of AMs inside the alveoli of the PCLS models, further Raman spectroscopic imaging experiments were carried out on unstained samples.

### 3.2 Raman spectroscopic characterization of the human ex vivo PCLS model

#### 3.2.1 Spectroscopic analysis of the tissue model environment

For in‐depth characterization of the macrophages in the alveoli by Raman spectroscopy, the spectroscopic background of the ex vivo tissue model was first examined in more detail. The spectroscopic background plays a decisive role in Raman measurements in tissues, as it can be expected to be present in varying proportions in each spectrum depending on the confocality of the setup. We recommend minimizing the background during sample preparation by avoiding the use of photoactive substances (e.g. phenol red) in the tissue culture medium. To preserve the 3D structure of the alveoli, the lung was perfused with agarose. The alveolar interior therefore showed an agarose spectrum with respective Raman bands at ∼740, ∼770, ∼845, ∼890, ∼970 and ∼1080 cm^−1^ (Figure S3). In addition to epithelial cells, the edges of the alveoli showed predominantly structure‐giving extracellular matrix including collagen fibres with typical Raman bands at ∼850, ∼940, ∼1005, ∼1245, ∼1325 and ∼1450 cm^−1^ (Figure S3). Both, the Raman spectra of the agarose and the collagen, were summarized as the general spectroscopic background of the ex vivo lung model, as they are to be expected in this form in all variations of the model. During the investigation of the immune cells in the tissue, these components were therefore largely excluded and not considered further.

#### 3.2.2 Raman spectroscopic imaging of AMs within a 3D human ex vivo PCLS model upon infection with SARS‐CoV‐2 variants delta and omicron

**FIGURE 2:**
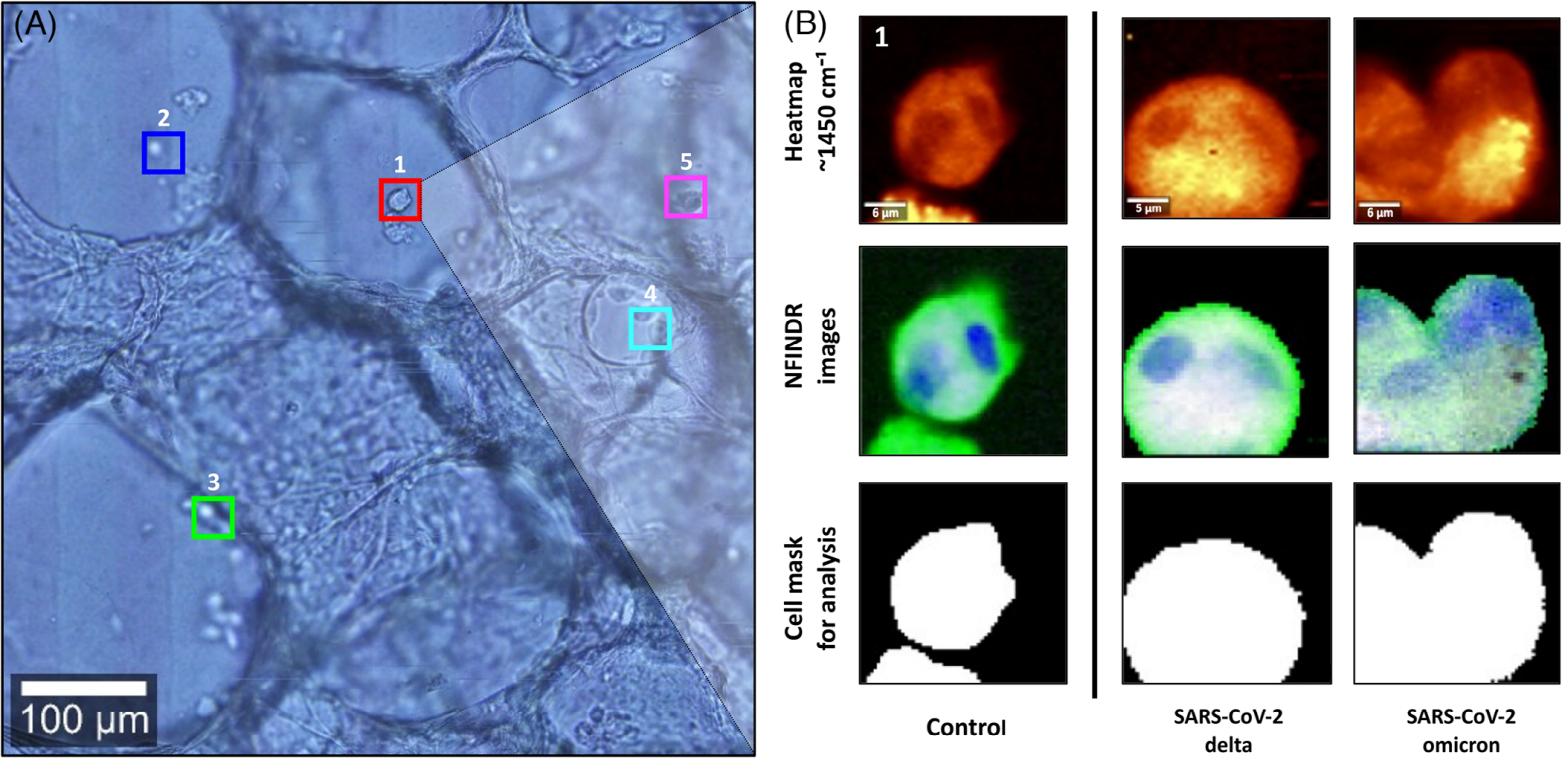
Raman spectroscopic imaging of alveolar macrophages in a human ex vivo precision‐cut lung slice (PCLS) model infected with SARS‐CoV‐2 variants. The figure shows (A) a bright field overview of lung alveoli and the position of alveolar macrophages within different Z levels of the uninfected control slice. Bright field images of the other conditions are shown in Figure S2. One exemplary cell from each treatment model is depicted in (B). Each cell was imaged individually and can be depicted as intensity distribution of the Raman band at 1450 cm^−1^ (CH deformation, top row). Furthermore, N‐FINDR images, indicating the spatial distribution of chemical components such as (phospho‐) lipids (green), nucleic acids (blue), and other chemical components summarized as cytoplasm (white) within the cell, or background spectra from the tissue model (black) are shown (middle row). Further images and corresponding Raman spectra are shown in Figures S4 and S5, respectively. Further spectral analysis of cell spectra was done after image segmentation, excluding the typical spectral background of the tissue model (bottom row).

Raman image scans from 15 human AMs in chemically fixed human ex vivo PCLSs (5 cells from each condition: uninfected controls, SARS‐CoV‐2 delta infected PCLS and SARS‐CoV‐2 omicron‐infected PCLS, respectively; cell positions inside PCLS models indicated in Figure S2) were analysed and visualized using the spectral unmixing algorithm N‐FINDR to depict the chemical composition of each cell (Figure 2, Figure S4). Thereby different unmixed Raman spectra were assigned to either background (as described in Subsection 3.2.1), nucleic acids, (phospho‐) lipids and cytoplasm including proteins and other biochemical substances within the cytosol (Figure S5). The assignment is used to visualize the spatial abundance of these components in colour‐coded Raman images to depict the intrinsic chemical contrast of the cells in a label‐free manner.

Overall, the distribution of chemical components within the cells can be seen best in the images of the uninfected control cells (Figure 2B). Cell nuclei were spotted and visualized in most of the cells within the intra‐alveolar space. AMs located at the borders of the alveoli (especially macrophages in the SARS‐CoV‐2 infection model) did not show as good resolved intracellular chemical compartments as in other cells.

**FIGURE 3:**
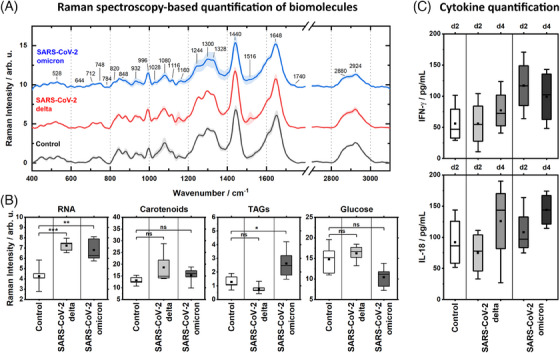
Quantification of cytokines and intracellular biomolecules indicating pro‐inflammatory activation. The figure shows (A) mean Raman spectra of alveolar macrophages (AMs) inside the uninfected control precision‐cut lung slice (PCLS) model (black) and both the PCLS models infected with either SARS‐CoV‐2 delta (red) or omicron (blue) variants (*n* = 5 AMs for each condition). Underneath (B), the Raman spectroscopy‐based quantification of intracellular RNA, carotenoids, triacyl glycerides (TAGs) and glucose on day 2 post‐infection is shown to better visualize shifts within the chemical composition of AMs upon viral infection. Significant differences using a one‐way ANOVA test are indicated with ***p < 0.001, **p < 0.01, *p < 0.05. Used Raman bands are listed in Figure S2. Cytokine quantification is shown in (C) and indicates the secretion of inflammatory cytokines on day 2 and day 4 post‐infection. Averages from three technical replicates (independent PCLSs from the same donor) are shown. Other cytokines and chemokine levels at day two and four are shown in Figure S8.

Mean Raman spectra of AMs (Figure 3A) from each experimental condition showed typical Raman bands of biological samples at ∼2800–3000 cm^−1^ (lipids, proteins), ∼1648 cm^−1^ (proteins), ∼1440 cm^−1^ (lipids, proteins), ∼1000 cm^−1^ (proteins), ∼1200–1350 cm^−1^ (proteins, DNA/RNA), ∼1000–1150 cm^−1^ (DNA/RNA, carbohydrates) and ∼750–800 cm^−1^ (DNA/RNA). Small Raman bands at ∼1150–1200 cm^−1^ and ∼1510–1520 cm^−1^ as well as at 1740 cm^−1^ can be assigned to intracellular carotenoids and triacyl glycerides (TAGs) stored in lipid droplets, respectively. It has to be noted that the intracellular carotenoids are not equally distributed in the AM. Thus, there Raman signature is not easily spotted in the mean spectra (Figure 3A), but the reader is referred to the computed difference Raman spectra in Figure S6A, which more precisely indicate the spectral differences among the cells of each experimental condition. To clearly localize spectral contributions of carotenoids within the AM, see also Figure S7 where a single unprocessed Raman spectrum from a single position is shown.

A quantitative analysis of spectral data (Figure 3B) showed significantly increased RNA levels in AMs infected with SARS‐CoV‐2 compared to the AMs of the uninfected control group. In addition, a trend toward slightly increased carotenoid levels in the infected cells was also recognizable.

The quantification of cytokines (Figure 3C) in the PCLS model infected with SARS‐CoV‐2 omicron revealed a slight increase in IFN‐y concentration as early as day 2 after infection. After four days of infection with either one of the two SARS‐CoV‐2 virus variants, increasing concentrations of IL‐18 became recognizable. In the PCLS model infected with SARS‐CoV‐2 delta, a slight increase in the IFN‐y level was recognizable on day 4 as well. Even if the changes in the cytokines were not significant, their development over time can probably be explained by the different reproduction times of the virus variants. AMs infected with SARS‐CoV‐2 omicron also showed a reduced intracellular amount of glucose and a simultaneously increased TAG level in the analysis of Raman spectra of the fixed PCLS model 2 days after infection (Figure 3B).

This could be interpreted as an indicator of the Warburg‐like switch—a metabolic adaptation in activated, pro‐inflammatory macrophages.^38^ Cells in the SARS‐CoV‐2 delta infected PCLS model did not show an immune response on day two after infection according to the cytokine levels measured. Thus, the glucose and TAG levels in the Raman analysis of these cells (Figure 3B) were similar to the levels in the uninfected control cells. Thus, the subtle differences detectable in the Raman spectra of the AMs could be interpreted in line with cytokine quantification.

#### 3.2.3 Macrophage phenotype classification using averaged Raman spectra and multivariate statistical analysis

Mean Raman spectra of AMs from uninfected and SARS‐CoV‐2 infected lung tissue were further statistically analysed using PCA followed by LDA.

**FIGURE 4:**
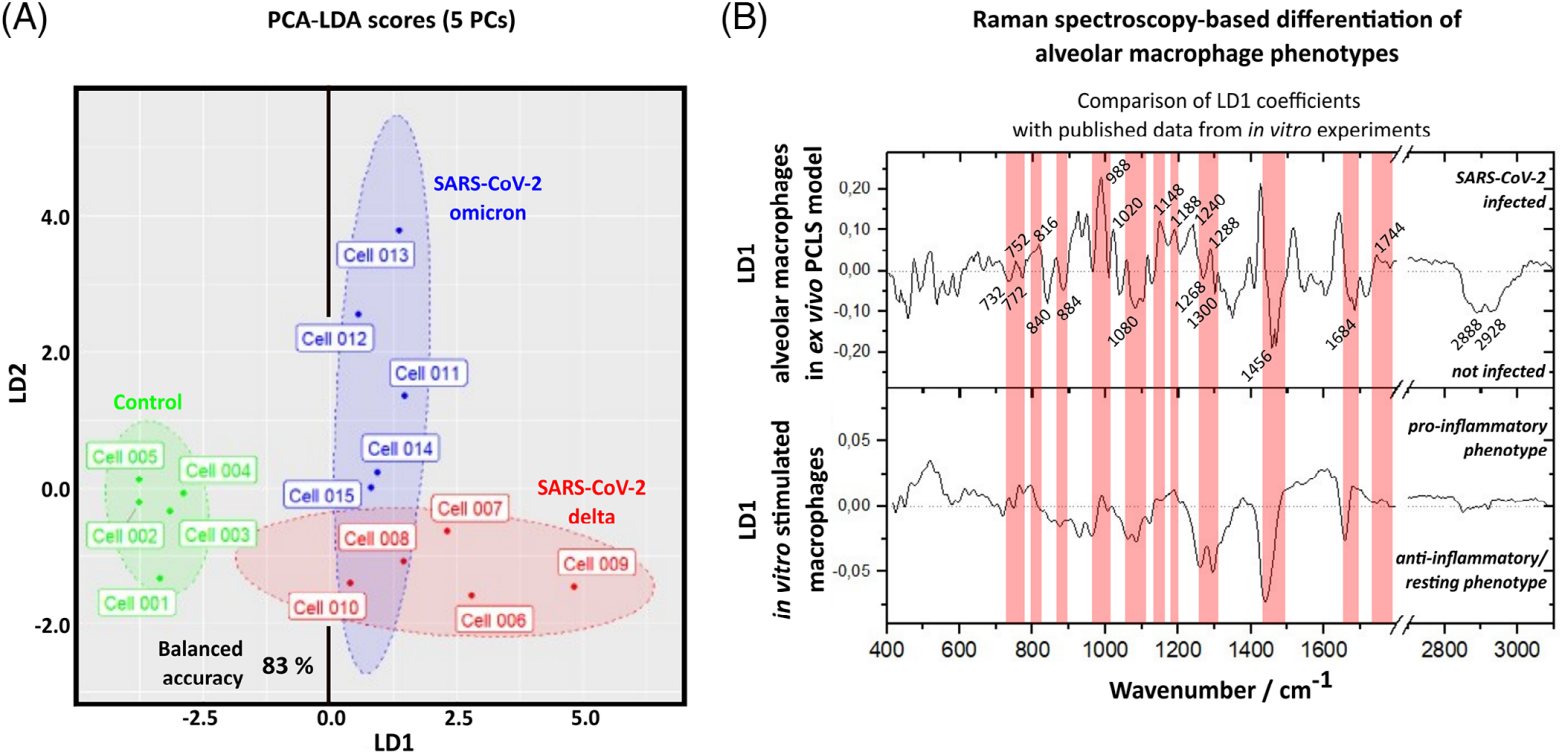
Principal component analysis‐linear discriminant analysis (PCA‐LDA) analysis of AMs in an ex vivo lung slice infection model. The figure shows (A) the 2D‐PCA‐LDA scores plot of the first two components used for discrimination. The LD1 loadings are shown in (B) in comparison to previously published data.^14^ Indicated spectral regions highlight similarities in LD1, which in the present study can be used to distinguish alveolar macrophages (AMs) from infected and uninfected precision‐cut lung slices. LD2 (Figure S6C) might be useful to discriminate alveolar macrophages depending on the virus variant infecting the PCLS, that is to differentiate infections with SARS‐CoV‐2 delta variant from those with SARS‐CoV‐2 omicron variant.

Thereby, a spectroscopic distinction between cells originating from uninfected and infected tissue slices could be made with a balanced accuracy of 83 % using “leave‐one‐cell from each condition‐out”—cross validation (Figure 4A, Figure S6B). Even the ex vivo infection with either SARS‐CoV‐2 delta variant or SARS‐CoV‐2 omicron variant was distinguishable. In comparison to previously published Raman spectroscopic data,^14^ where pro‐ or anti‐inflammatory functional macrophage phenotypes were induced in isolated macrophages in vitro, especially spectral features assigned to DNA/RNA, fatty acids and carbohydrates showed similarities (Figure 4B). This suggests that the SARS‐CoV‐2 infected AMs develop a more pro‐inflammatory phenotype compared to the uninfected AMs according to spectroscopic analysis.

## 4 DISCUSSION

### 4.1 Raman spectroscopy indicates changes in chemical composition of AMs upon ex vivo infection with SARS‐CoV‐2 variants

AMs play a crucial role in infections of the pulmonary tract, as they can both initiate an initial immune response to the pathogen and contribute to the resolution and clearance of the infection. In the case of infection with SARS‐CoV‐2 variants, immunofluorescence staining (Figure 1B) showed that the viral spike protein also co‐localized with AMs in the PCLS infection models, indicating the presence of viral material within the AM. Upon closer inspection of the Raman microscopic data, small Raman spectroscopic differences became apparent, indicating significantly increased RNA levels in AM in infected tissue slices. This could be explained with the accumulation of phagocytized viral material. SARS‐CoV‐2 virus directly infects epithelial cells (as seen in Figure 1B), replicates inside them and ultimately leads to apoptosis of the epithelial cells. AM clear cell debris with virus particles leading to AM with increased RNA content at day 2 after infection. Some studies also suggest direct viral uptake and even potential active viral replication in M1 AM.^6^, ^7^, ^39^ However, the completion of the viral life cycle in macrophages is still up for debate^40^, ^41^ and other studies^42^ state that SARS‐CoV‐2 virus is not replicating in macrophages. In own unpublished cell experiments, we also could not achieve infection with and active replication of SARS‐CoV‐2 in macrophages. Within the current study, we cannot answer the question whether the increased RNA content originates from direct virus uptake or from phagocytosing infected epithelial cells, neither can we quantify extend of phagocytosis of the individual AM. A larger cell‐to‐cell‐variation among the AM from infected tissue slices compared to AM from uninfected control slices (visible in Figure 4A), points to a slightly larger intercellular heterogeneity in AM from infected lung slices, which—however—should not be overinterpreted with the currently small sample size. Those questions should be addressed in further follow‐up studies and supported with appropriate fluorescence co‐staining experiments.

Especially in the PCLS model infected with SARS‐CoV‐2 omicron, a significant immune response was already recognizable on day 2 after infection based on the measured cytokine levels. This also matches the reproduction times published for the omicron variant, which are shorter than the reproduction times of the delta variant.^17^, ^43^ The immune response as part of the AMs was also detected by Raman spectroscopy. Overall spectral difference between AMs from infected tissue slices as compared to AMs from uninfected control slices point to a different activation state/phenotype of the AMs which can be induced by phagocytosis, but also by reaction to infection‐induced cytokines and chemokines in the lung tissue. Based on the differences in spectral features associated with fatty acids and glucose, AMs showed an increased level of intracellular fatty acids (TAGs) and a decreased level of glucose on day 2 post‐infection with SARS‐CoV‐2 omicron. It has to be noted that the glucose discussed here is thought to originate from inside the cells as part of their metabolism and not considers extracellular glucose in the alveolar space (which are known to be low). Increased glucose levels within AM suggest a metabolic reprogramming of the cells and is in agreement with the known pro‐inflammatory phenotype switch of AMs enhancing the glycolytic pathway while uncoupling oxidative phosphorylation (OXPHOS) in mitochondria (Warburg‐like metabolic changes in M1‐like macrophages).^38^, ^44^, ^45^ This impairment of mitochondrial OXPHOS promotes the formation of reactive oxygen species (ROS) and the associated oxidative stress, against which the cells protect themselves primarily with free radical scavenging antioxidants such as carotenoids.^46^, ^47^, ^48^

### 4.2 Raman spectroscopic results are consistent with previously published results and reflect metabolic adaptations of the cells’ phenotypes to infectious situations

Recent publications have shown that Raman spectroscopy in combination with multivariate statistical analysis methods is a useful tool to distinguish different functional phenotypes of macrophages in vitro.^13^ Pro‐ and anti‐inflammatory phenotypes were mainly distinguishable from each other based on differences in the spectral signatures of fatty acids, DNA/RNA and carbohydrates with a balanced accuracy of >80%.^14^ In this study, the same statistical analysis method was used and led to a distinction between AMs inside uninfected control and virally infected ex vivo human PCLS models with an accuracy of comparable 83%. Even though AMs were measured in a more natural‐like environment, the LDA loadings had many similarities in the above‐mentioned spectral signatures indicating the presence of M1‐like activated macrophages after a broader immune activation in the ex vivo human PCLS model infected with SARS‐CoV‐2. This underlines the reproducibility and validity of the results and highlights the potential of Raman spectroscopy as a tool for monitoring and analysing changes in the chemical composition of immune cells upon infectious events (immunometabolic modulations).

### 4.3 Limitations of the study

The main limitation of the study is the small sample size. Healthy lung tissue from only one donor was used. During preparation, the lung tissue was separated into several precision cut lung slices which were treated, that is infected, separately and independently from each other. Nevertheless, this means, while technical replicates were used in this study, only one biological independent donor was characterized. This means, no conclusions can be drawn on the influence of donor specific factors, such as age, prior exposures, and potential epigenetic modifications. We are aware that these factors might influence AM phenotype or alter macrophage function, activation states, and metabolic profiles,^49^ thereby potentially impacting the interpretation of spectroscopic data. Data from this study show infection‐induced changes in AMs from a healthy, young (35 years old), male donor. Unfortunately, access to lung tissue from healthy donors is very limited and not routinely available for research. Thus, the sample size could not be increased within this study. Nevertheless, the described Raman‐based method is generally applicable to other tissue sections as well.

To ensure an unbiased evaluation of the model's performance on the small data set and mitigating the risk of overfitting, the PCA‐LDA classification model utilized a “Leave‐One‐Cell‐Out‐From‐Each‐Condition” cross‐validation strategy.

While it is known that AMs exhibit high functional plasticity and can display mixed or transitional phenotypes depending on their environment, we could not sufficiently explore these nuances within our study. However, this would be of interest to follow up in subsequent studies.

In this study, we compared the spectral features of activated AMs to the ones of monocyte‐derived macrophages. We are aware that a comparison to a direct model with AM would be more suitable. However, it is not easy to collect from healthy individuals as they usually do not receive a bronchoalveolar lavage (BAL). Thus, we did not receive sufficient cells from healthy donors. Furthermore, a SARS‐CoV‐2 infection model in AMs has to be carefully planned. As SARS‐CoV‐2 is not (easily) replicating in macrophages, a co‐infection model would need to be set up to mimic the effects taking place in the complex human lung.

## 5 CONCLUSION

In this study, Raman spectroscopy was used to investigate the cellular response of AMs in an ex vivo human PCLS model to infection with either SARS‐CoV‐2 delta or omicron variants. The PCLS model was optimized for Raman spectroscopic investigations and spectroscopically characterized to ensure successful investigations of AMs in their natural tissue composites. False‐colour Raman images of AMs were obtained without any labelling and compared to immunofluorescence‐stained samples. AM Raman spectra showed characteristic spectral features of AMs and changes in the chemical composition of the cells after infection with SARS‐CoV‐2 variants could be tracked spectroscopically. In addition to a significantly increased intracellular RNA level, trends in carotenoid, TAG and glucose levels were also identified, which could be interpreted in accordance with data on cytokine quantification and respective literature. Based on this, a PCA‐LDA classification model was trained to distinguish between uninfected and SARS‐CoV‐2 infected AMs with a robust accuracy of 83%. Even a trend to distinguish a SARS‐CoV‐2 delta mediated infection from an infection initiated by the omicron variant was found. However, in order to be sure that the spectral differences are due to the viral material inside the cells and not just due to different viral replication stages, further, more in‐depth investigations are necessary. Nevertheless, the Raman data agreed with results from previously published in vitro experiments, underlining that Raman spectroscopy is a promising technology in immunology and clinical diagnostics to study the cellular immune response to infectious diseases.

## AUTHOR CONTRIBUTIONS

Max Naumann, Ute Neugebauer and Stefanie Deinhardt‐Emmer conceived and designed the study. Max Naumann, Franziska Hornung, Simone Eiserloh, Tim Sandhaus and Astrid Tannert developed the methodology, contributed to biological sample preparation and performed the experiments. Max Naumann and Rustam R. Guliev analysed the data and developed scripts. Max Naumann, Ute Neugebauer and Stefanie Deinhardt‐Emmer discussed results. Max Naumann wrote the manuscript. Critical feedback and research supervision were provided by Stefanie Deinhardt‐Emmer and Ute Neugebauer. All authors have read, contributed and agreed to the published version of the manuscript.

## CONFLICT OF INTEREST STATEMENT

The authors declare no conflicts of interest.

## ETHICS STATEMENT

Experiments utilized lung tissue from one healthy donor with no known underlying diseases after informed written consent. This research was approved by the local ethics board of the Jena University Hospital (approval numbers: 2018‐1263, 2020‐1894 and 2020‐1773).

## Supporting information



Supporting Information

## Data Availability

All data are available upon request.
