# Supplementary material for: Investigating alveolar macrophages in an human ex vivo precision‐cut lung slice model of SARS‐CoV‐2 infection using Raman spectroscopy—A case study
Source: Clin Transl Med. 2025 Aug 31;15(9):e70453. doi: 10.1002/ctm2.70453 (PMC12399784; doi:10.1002/ctm2.70453)
Supplement: Supplementary file 1 — Supporting Information [file CTM2-15-e70453-s001.docx]

Supplementary Information on

Investigating Alveolar Macrophages in a Human *Ex- Vivo* Precision-cut Lung Slice Model of SARS-CoV-2 Infection Using Raman Spectroscopy

Max Naumann, Franziska Hornung, Simone Eiserloh, Astrid Tannert, Antje Häder, Rustam R. Guliev, Tim Sandhaus, Stefanie Deinhardt-Emmer and Ute Neugebauer

**Statistical analysis of spectroscopic data**

***Supplemental Table S1: Overview of measured Raman data, that was used for data analysis.*** *The table summarizes the number of measured alveolar macrophages as well as the corresponding number of spectra before and after preprocessing. Removing non-cellular background and environmental background arising from the PCLS model accounts for the high reduction of number of spectra after preprocessing.*

| **Condition** | **Number of images recorded** | **Number of images after preprocessing** | **Number of spectra recorded** | **Number of spectra after preprocessing** |
| --- | --- | --- | --- | --- |
| Control | 5 | 5 | 18,000 | 7,250 |
| SARS-CoV-2 delta | 5 | 5 | 10,000 | 5,097 |
| SARS-CoV-2 omicron | 5 | 5 | 18,000 | 9,847 |
| **∑** | **15** | **15** | **46,000** | **22,194** |

***Supplemental Table S2: Raman bands used for quantitative analysis.*** *The table lists the wavenumber ranges used to generate the box plots in Figure 3B, main manuscript. The spectral regions as well as the Raman bands annotated in the spectra were selected based on previously published data ^1-6^ (Ref. [31–36] in main manuscript).*

| **Chemical components** | **Raman bands** |
| --- | --- |
| RNA  Carotenoids  TAGs  Glucose | 780~790 cm^-1^, 805~815 cm^-1^, 980~990 cm^-1^  990~1000 cm^-1^, 1145~1155 cm^-1^, 1510~1520 cm^-1^  1730~1750 cm^-1^  1050~1070 cm^-1^, 1115~1125 cm^-1^ |

References;
1. A. Rygula, K. Majzner, K. M. Marzec, A. Kaczor, M. Pilarczyk and M. Baranska, *Journal of Raman Spectroscopy*, 2013, **44**, 1061-1076.

2. K. Czamara, K. Majzner, M. Z. Pacia, K. Kochan, A. Kaczor and M. Baranska, *Journal of Raman Spectroscopy*, 2014, **46**, 4-20.

3. E. Wiercigroch, E. Szafraniec, K. Czamara, M. Z. Pacia, K. Majzner, K. Kochan, A. Kaczor, M. Baranska and K. Malek, *Spectrochim Acta A Mol Biomol Spectrosc*, 2017, **185**, 317-335.

4. M. Dudek, G. Zajac, E. Szafraniec, E. Wiercigroch, S. Tott, K. Malek, A. Kaczor and M. Baranska, *Spectrochim Acta A Mol Biomol Spectrosc*, 2019, **206**, 597-612.

5. J. M. Benevides, S. A. Overman and G. J. Thomas, *Journal of Raman Spectroscopy*, 2005, **36**, 279-299.

6. G. Pezzotti, *Journal of Raman Spectroscopy*, 2021, **52**, 2348-2443.

**Raman spectroscopic imaging of alveolar macrophages**


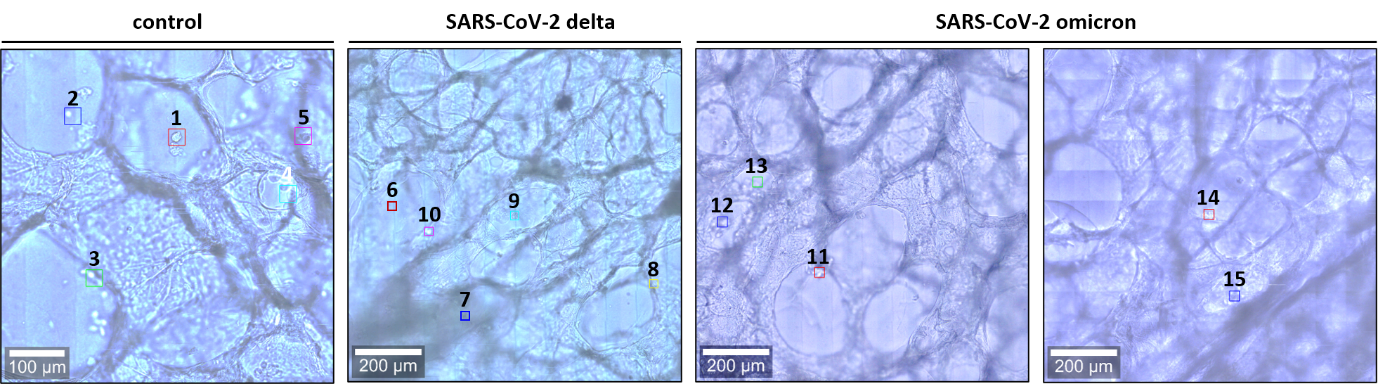


***Supplemental Figure S2: Bright field images of alveolar macrophages in PCLS models.*** *The bright field images indicate the precise position of the 15 alveolar macrophages within the 3D structure of the respective PCLS models investigated with Raman spectroscopy.*

*
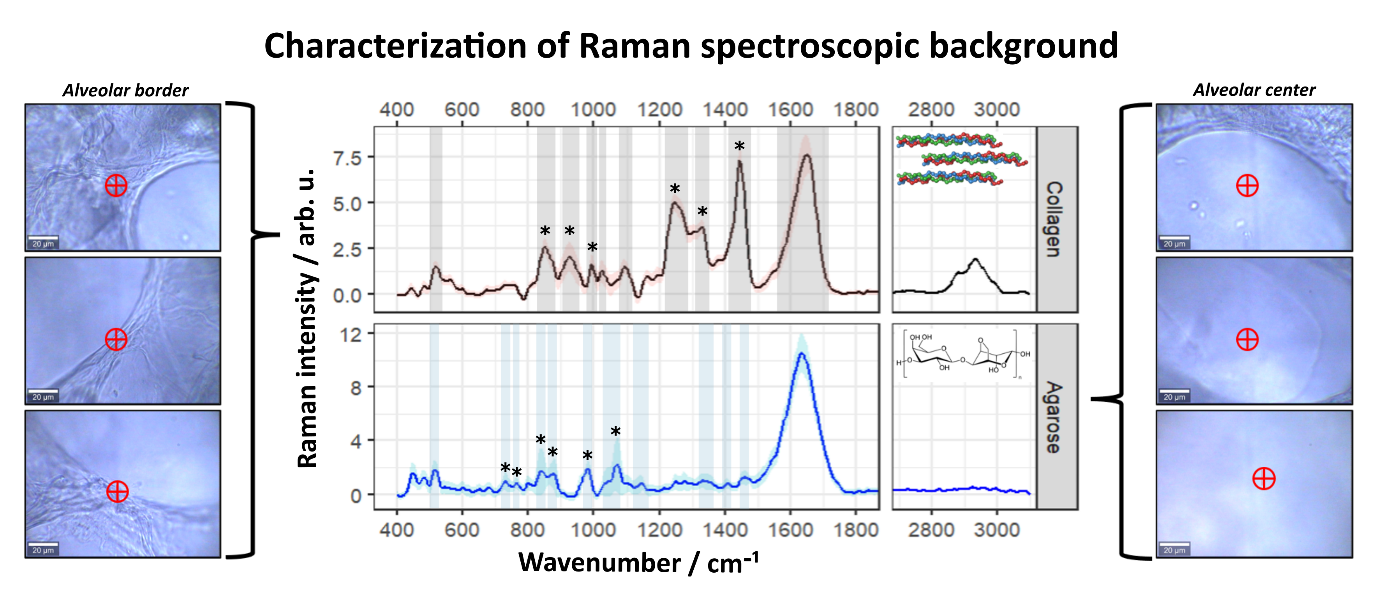
*

***Supplemental Figure S3: Characterization of Raman spectroscopic background of the* ex vivo *PCLS model.*** *The scheme shows mean Raman spectra recorded at indicated positions within the* ex vivo *lung slice model within as well as at the borders of the alveoli. These typical spectra were considered as spectroscopic background and excluded throughout the Raman-based analysis of alveolar macrophages. Asterisks highlight the spectral position of typical Raman bands for both collagen fibers (~850 cm^-1^, ~940 cm^-1^, ~1005 cm^-1^, ~1245 cm^-1^, ~1325 cm^-1^ and ~1450 cm^-1^) and agarose (~740 cm^-1^, ~770 cm^-1^, ~845 cm^-1^, ~890 cm^-1^, ~970 cm^-1^ and ~1080 cm^-1^).*

| **Alveolar macrophages inside the control PCLS model** |
| --- |
| 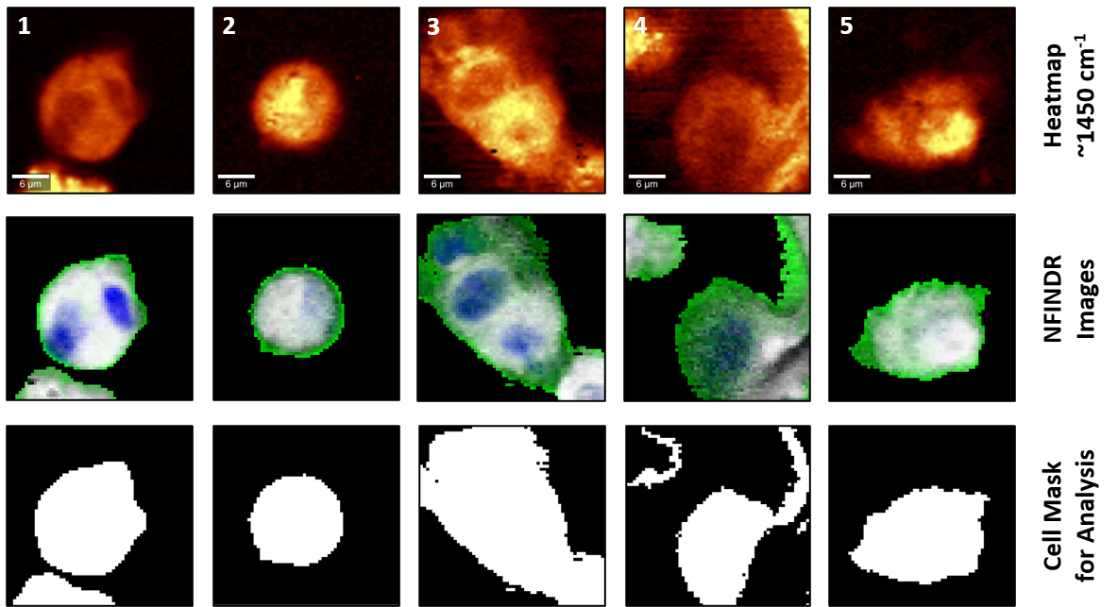 |
| **Alveolar macrophages inside the SARS-CoV-2 delta infected PCLS model** |
| 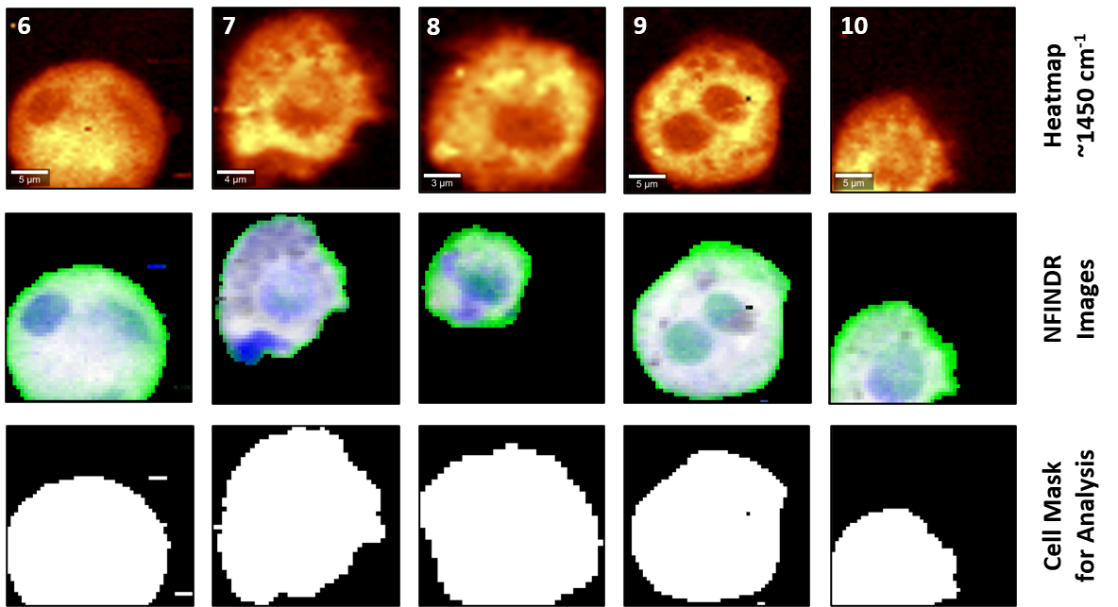 |
| **Alveolar macrophages inside the SARS-CoV-2 omicron infected PCLS model** |
| 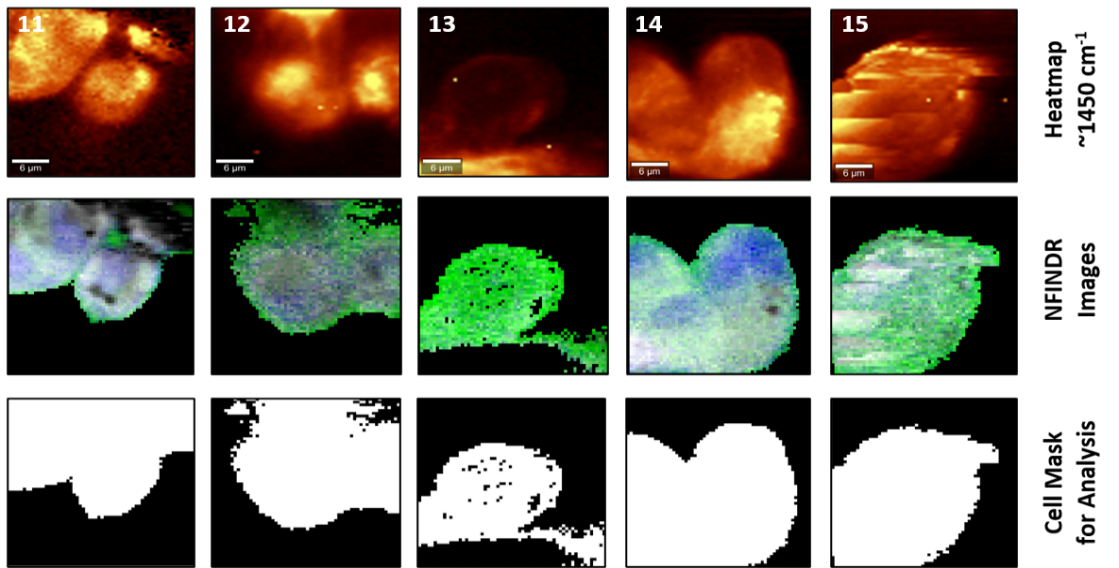 |

***Supplemental Figure S4: Raman spectroscopic images of AMs in PCLS models.*** *The scheme depicts the Raman images of all 15 AMs inside the respective PCLS models as Raman intensity images at ~1450/50 cm^-1^ (Heatmap top row in each panel), false color NFINDR images (middle rows) and binary masks of spectra assigned to AMs (bottom row), that were further used for spectral analysis. Respective N-FINDR endmember spectra are shown in Supplementary Figures S5.*


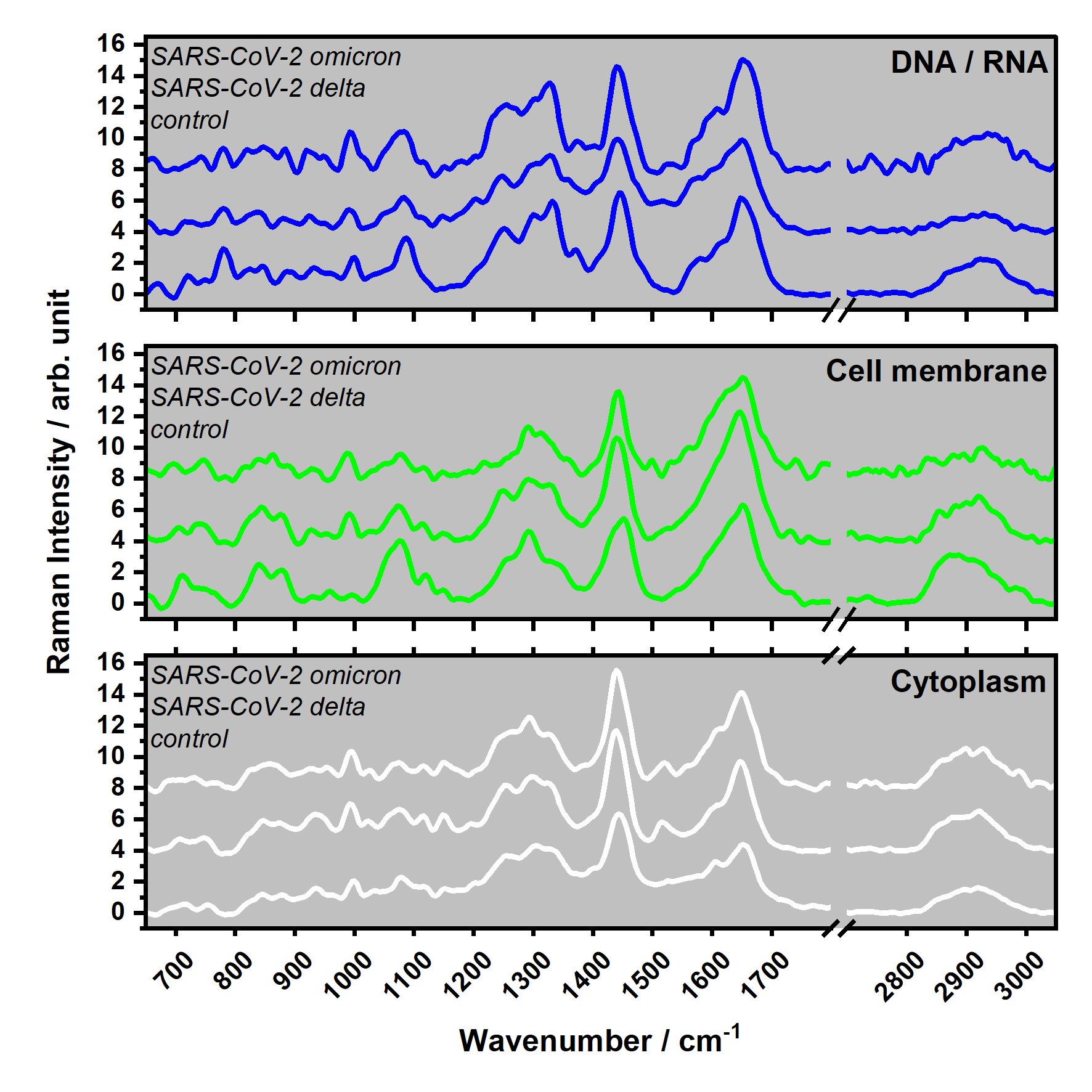


***Supplemental Figure S5: Endmember spectra of the NFINDR images shown in Suppl. Figure S4.*** *The figure depicts endmember spectra found after spectral unmixing using the NFINDR algorithm. Spectra were assigned to cell nuclei (blue: DNA/ RNA), cell membrane (green) consisting of (Phospho-) lipids and proteins and the cytoplasm (white) with all its biochemical ingredients. Color code corresponds to the one used in Figure 2B main manuscript and Supplementary Figure S4.*

**Multivariate statistical analysis of averaged Raman spectra**


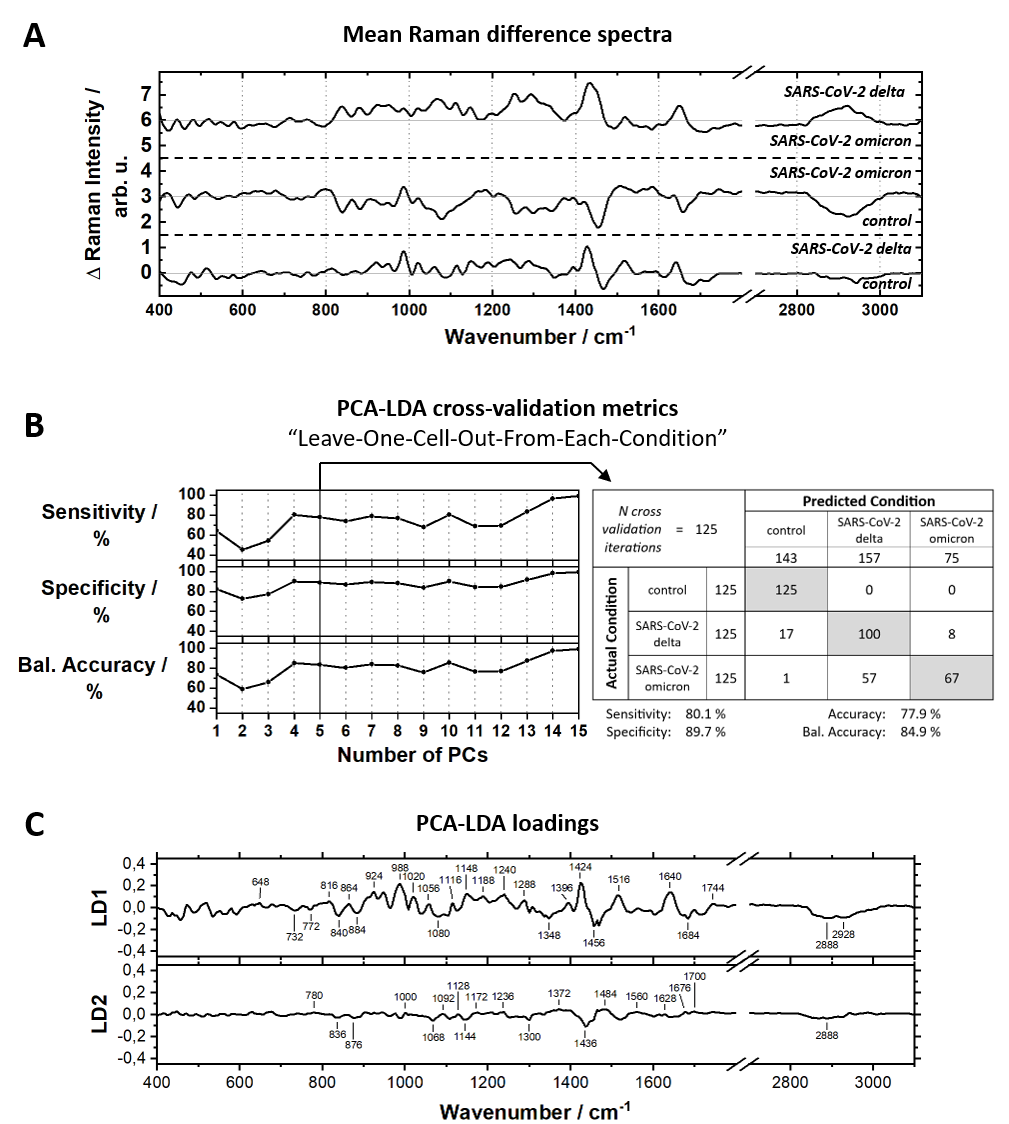


***Supplemental Figure S6: Analysis of mean Raman spectroscopic differences and binary PCA-LDA model (trained on averaged spectra per cell) identifying AMs in SARS-CoV-2 infected PCLS models.*** *The figure depicts (A) the mean Raman difference spectra indicating spectral differences between AMs inside the control PCLS model and both the PCLS models either infected with SARS-CoV-2 delta or omicron variants. (B) A PCA-LDA model was trained using averaged Raman spectra per cell. The plot on the left shows PCA-LDA performance metrics based on combined test predictions from 125 "Leave-One-Cell-Out-From-Each-Condition" cross-validation cycles. The vertical line indicates the selected model using 5 principal components (PCs). The corresponding confusion matrix is depicted on the right side. (C) Linear discriminant loadings (PCA-LDA model with 5 PCs) that are used for discrimination are shown.*

*
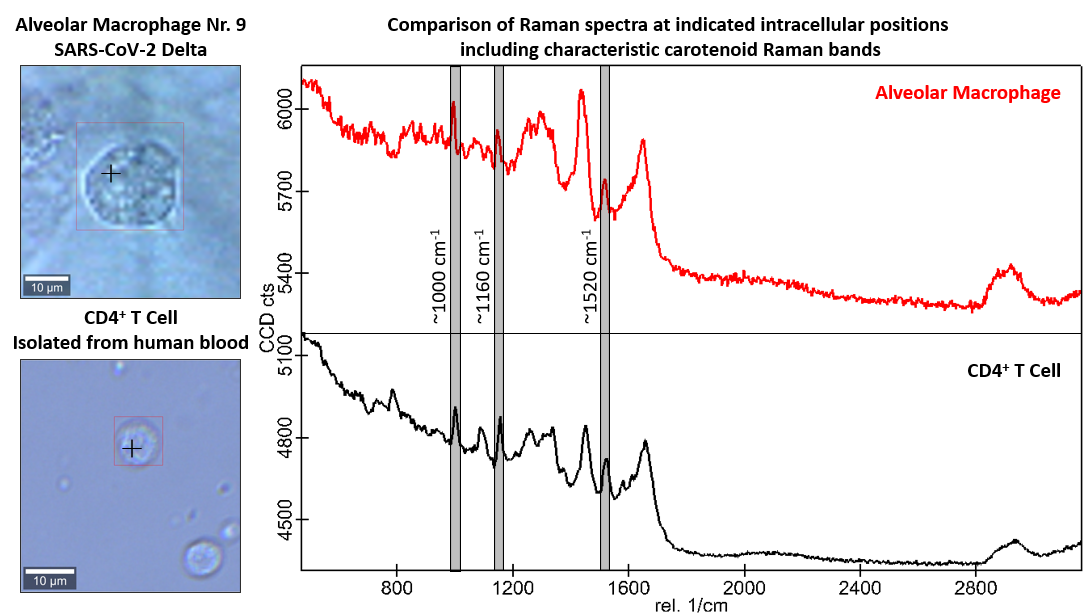
*

***Supplemental Figure S7:*** *Raman spectral features of carotenoids. Exemplary unprocessed Raman raw spectra at indicated positions within an alveolar macrophage (AM 9) from SARS-CoV-2 Delta infected PCLS and within a CD4+ T cell isolated from human blood are shown in comparison. Both spectra show characteristic Raman bands of carotenoids with high agreement.*

***
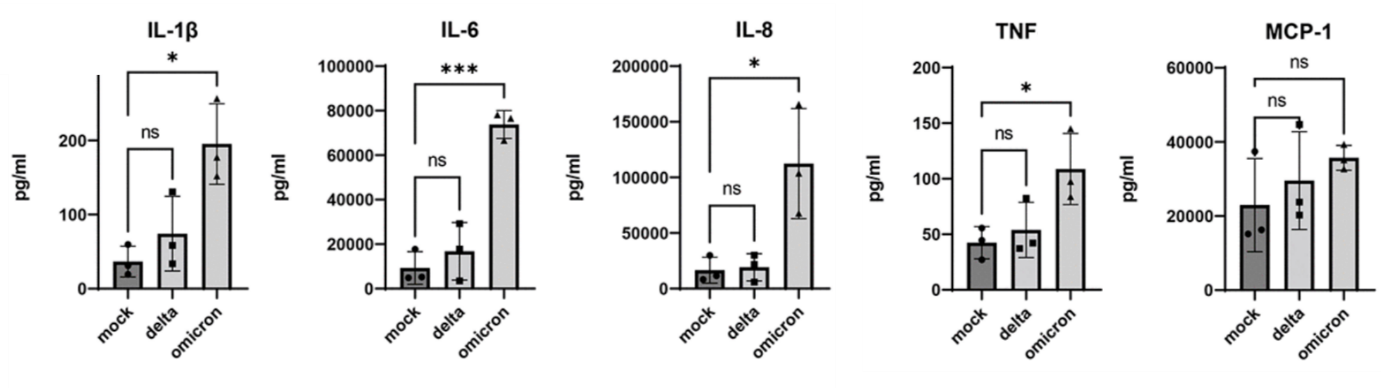
***

***Supplemental Figure S8: Quantification of cytokines and chemokines in the* ex vivo *PCLS model.*** *Inflammatory cytokines and chemokines in the supernatant of the infected lung slices revealed that infection with the omicron variant triggered an increased secretion of IL-1β, IL-6, IL-8, and TNF. While values for MCP-1 were on average highest in omicron slices, they did not reach statistical significance. P calculated by one-way ANOVA with Multiple comparisons, *p < 0.01, **p < 0.05, ***p < 0.001. (Figure from Hornung et al. Aging (Albany NY). 2023 Dec 12; 15:13593-13607 .* <https://doi.org/10.18632/aging.205297>*)*
